# Lipoprotein(a): Role in atherosclerosis and new treatment options

**DOI:** 10.17305/bb.2023.8992

**Published:** 2023-08-01

**Authors:** Dragana Tomic Naglic, Mia Manojlovic, Sladjana Pejakovic, Kristina Stepanovic, Jovana Prodanovic Simeunovic

**Affiliations:** 1 Faculty of Medicine in Novi Sad, University of Novi Sad, Novi Sad, Serbia; 2 Clinic for Endocrinology, Diabetes and Metabolic Disorders, Clinical Center of Vojvodina, Novi Sad, Serbia

**Keywords:** Lipoprotein(a) (Lp(a)), lipids, atherosclerosis, major adverse cardiovascular events (MACE), cardiovascular (CV) diseases, cardiovascular prevention

## Abstract

Atherosclerosis is a chronic process characterized by inflammation and the progressive accumulation of inflammatory cells and lipids in the blood vessel wall, resulting in narrowing of the blood vessel’s circumference. Treatment of people with dyslipidemia aims to reduce the risk of developing atherosclerotic disease and prevent major adverse cardiovascular events (MACE). The results of previous studies indicated that lipoprotein(a) (Lp(a)) is a critical causal factor in the estimated risk of developing a cardiovascular (CV) incident even after achieving desirable low-density lipoprotein (LDL) cholesterol levels. Lp(a) is a low-density lipoprotein particle, like LDL cholesterol. The levels of Lp(a) in plasma are genetically determined. Lp(a) catabolism is still controversial. The pathogenic potential of Lp(a) can be divided into three categories: promotion of plaque formation, thrombogenicity, and proinflammatory effects. Lp(a) levels above the 75th percentile reduced the risk of aortic valve stenosis and myocardial infarction, whereas higher levels (above 90th percentile) were associated with an increased risk of heart failure. However, no hypolipidemic agents have been approved for targeted use in patients with high Lp(a) levels. There are insufficient randomized-controlled trials assessing CV outcomes that would support the evidence that current treatment options, which effectively lower Lp(a) levels, also effectively prevent CV event. However, according to some studies, there is strong evidence that better CV outcome is one of the benefits of such therapy. The results of ongoing clinical trials are eagerly awaited.

## Introduction

Atherosclerosis is a chronic process characterized by inflammation and progressive growth of inflammatory cells and lipids in the blood vessel wall, which results in narrowing of the blood vessel circumference. In case of erosion or rupture of the plaque, acute, subacute, or chronic process of thrombosis can occur as a complication of atherosclerosis. Among the various risk factors, established lipid parameters, such as elevated low-density lipoprotein (LDL) cholesterol, elevated triglycerides, and also reduced high-density lipoprotein (HDL) levels, affect the course of accelerated atherosclerosis. In addition to the aforementioned lipid parameters, the course and rate of atherosclerotic changes are influenced by genetic (familial dyslipidemia or familial predisposition to diabetes) and behavioral characteristics (diet, sedentary lifestyle, and smoking habits). Recent research indicates that, in addition to LDL cholesterol, all particles rich in apolipoprotein B100 (apo B-100), including very low-density lipoprotein (VLDL) cholesterol and intermediate-density lipoprotein cholesterol, exhibit the greatest atherogenicity [1].

The goal of treating people with dyslipidemia is to minimize the risk of developing atherosclerotic disease and to prevent major adverse cardiovascular events (MACE). This goal has been set thanks to numerous randomized clinical trials with cardiovascular (CV) outcomes demonstrating the beneficial effects of statins in primary and secondary prevention [2–12].

However, Makover et al. [13] highlighted that the outcomes of other atherosclerosis-related diseases have been neglected in previous intervention trials. This group of diseases includes peripheral arterial disease, dementia, heart failure, renal failure, renal artery stenosis, carotid artery stenosis or embolism, arterial hypertension, aortic valve disease, and erectile dysfunction. The same author suggests that in future studies and daily clinical work, priority should be given to slowing down the above diseases.

According to established guidelines, statins are still the first line of therapy in the secondary prevention of CV events. It is also recommended to use high-potency statins in high doses that are well tolerated by the patient until target LDL cholesterol levels are reached. In contrast, in primary prevention in individuals with a familial form of hyperlipoproteinemia or an estimated 10-year risk of CV disease (CVD) in the field of accelerated atherosclerotic cardiovascular disease (ASCVD), if LDL cholesterol: levels below 1.4 mmol/L are not achieved in individuals at very high or high risk, the target LDL cholesterol level is below 1.8 mmol/L, whereas in apparently healthy individuals, the target value is below 2.6 mmol/L, than the possibility of introducing ezetimibe should be considered. If the target is not reached, the next step is the addition of proprotein convertase subtilisin/kexin type 9 (PCSK-9) inhibitors [14, 15].

In addition to the well-documented reduction of atherogenic lipoprotein particles level, statins are the first line of therapy due to their pleiotropic effects, which include an anti-inflammatory effect (reduction of macrophage chemotaxis and adhesion, and suppression of T cell activity), an antioxidant effect (reduction of free radicals, reduction of nicotinamide adenine dinucleotide phosphate [NADPH] oxidase), suppression of angiogenesis (by decreasing the level of platelet-derived growth factor and fibroblast growth factor, preventing smooth muscle cell hypertrophy in blood vessel), antiplatelet effect (by stimulating the release of nitric oxide [NO], stimulating fibrinolysis, decreasing the aggregability of platelets, and increasing the expression of thrombomodulin), and improving endothelial dysfunction [16].

The results of the JUPITER study indicated that Lp(a) is a critical determinant of residual risk for developing a CV event, even after achieving desirable LDL cholesterol levels. In particular, the use of rosuvastatin during the two-year conduct of the JUPITER trial demonstrated the benefit of statin therapy in the primary prevention of CV events, even in a low-risk population, such as nonsmokers, women, people with normal diet, and people without metabolic syndrome [17].

In the subsequent follow-up, during another two years, subjects from the JUPITER study were analyzed, and Khera et al. used composite CV events (myocardial infarction, ischemic stroke, hospitalization for unstable angina, coronary revascularization, and CV mortality) as the primary goal, as well as total mortality (total mortality rate) to analyze residual risk. During these periods, all subjects were taking rosuvastatin. The results of the study clearly indicated that in persons who reached the target levels of LDL cholesterol, a strong predictor of morbidity and mortality is precisely the level of Lp(a) [18].

## Lipoprotein(a) metabolism

Lipoprotein(a) (Lp(a)) is a low-density lipoprotein particle, like LDL cholesterol. Berg [19] discovered this particle exactly 60 years ago. Namely, like LDL cholesterol, it consists of a solid lipid core of cholesterol esters with phospholipids and non-esterified cholesterol on the surface. What distinguishes these two particles is the presence of modified apoprotein B, i.e., apoprotein B is binded with covalent disulphide bridge to apoprotein(a) (apo(a)) into the composition of Lp(a) lipoprotein [20]. The synthesis of apo(a) occurs exclusively in the liver under the influence of the *LPA* gene, which is located on the long arm of the 6th chromosome at positions 26 and 27 (6q26-27) [21].

The binding of apo(a) to the LDL cholesterol particle occurs in the extracellular space of hepatocytes, most likely partially in the interstitium of the liver and partially in the plasma. There is also evidence of binding of apo(a) to VLDL particles that can be transformed into Lp(a). The formation of Lp(a) lipoproteins also takes place in the bloodstream, through lipolysis under the action of lipoprotein lipase when triglyceridemic remnant particles bound to apo(a) are formed, allowing recycling, i.e., the reuse of previously synthesized and used apo(a) for the production of Lp(a) lipoproteins in the interstitium of the liver [22, 23]. These findings are significant because they open new possibilities for therapeutic interventions against Lp(a) hyperlipoproteinemia.

Lp(a) catabolism is still controversial. Catabolism is thought to occur partially in the liver and partially in the kidneys, spleen, and muscle tissue. The elimination half-time of Lp(a) is twice as long as that of LDL cholesterol, averaging four days. The prolonged elimination time is probably due to the complex structure of Lp(a) particles and the need to degrade both apo(a) and apoB-100 and oxidized phospholipid particles. Although the catabolism of Lp(a) particles remains largely unclear, recent research data in an animal model show that adequate catabolism of Lp(a) requires the physiological activity of at least five groups of receptors: plasminogen, lecithin, classic lipoprotein receptors, scavenger receptors, and Toll-like receptors [24].

The level of Lp(a) in plasma is genetically determined. However, some conditions can affect the fluctuation of this lipoprotein level. These include acute inflammatory diseases, pregnancy, liver and kidney diseases, untreated hypothyroidism, acromegaly, and myocardial infarction. These conditions can lead to a transient increase in Lp(a) levels. After the management of the mentioned conditions, Lp(a) values return to the reference range [25–35].

## The pathogenic potential of lipoprotein(a)

The pathogenic potential of Lp(a) in terms of the development of CVD lies not only in its atherogenic potential but numerous other mechanisms are also known. The mechanism by which Lp(a) causes complications in the form of accelerated atherosclerosis and aortic valvular calcification is most likely fundamentally similar. Comparing the histological slides of both entities, the findings are almost similar and correspond to the accumulation of lipids in the intima media of blood vessels, abnormal infiltration of inflammatory cells, and advanced fibrosis. Moreover, the common term for both processes is calcification of the blood vessel wall [36].

The pathogenic potential of Lp(a) can be divided into three categories: promotion of plaque formation, thrombogenicity, and inflammatory effect [37]. As mentioned previously, Lp(a) contains mutated apoB100, associated with apo(a) by a disulphide bridge, which decreases the affinity of these particles for LDL receptors in the liver and prevents their utilization in hepatocytes. Apo(a) has an increased affinity for scavenger cells in the blood vessel wall. It quickly penetrates macrophages, forms foam cells, and activates a cascade of inflammatory processes that contribute to the acceleration of atherosclerosis [38]. Lp(a) is the carrier of the activator of phospholipase A2, which is responsible for the degradation of oxidized fatty acids. In this way, the content of short-chain fatty acids and lysolecithin in serum increases and enhances the lipotoxic effect. The oxidized Lp(a) molecule contains an oxidized LDL particle, which is immunogenic itself, and potentiates the production of autoantibodies against Lp(a) particles. In all studies performed, the mere existence of these antibodies was observed as an integral component of the atheroma plaque. The thrombogenicity of Lp(a) is reflected in the inhibition of fibrinolysis [37].

There is homology in the chemical composition of apo(a) and plasminogen, but apo(a) does not have the plasminogen enzymatic activity. Because of the structural similarity, the production of active plasminogen is inhibited and the homeostasis of thrombogenic and fibrinolytic activity is disrupted, with inhibition of fibrinolysis and enhancement of thrombogenesis. The fibrinolytic system recognizes apo(a) as plasminogen and thus reduces the production of plasminogen. However, blood coagulability is activated, in order to maintain physiological equilibrium [39].

Lp(a) is liable to oxidative modification, resulting in the formation of the oxidation-specific epitope. Oxidation-specific epitope is located on oxidized LDL, cell debris, apoptotic cells, and modified proteins in the blood vessel wall that accumulate in response to hypercholesterolemia and elicit a strong proinflammatory response (through monocyte/macrophage chemotaxis) as disease-specific antigens [37].

This molecule is known to bind to fibrin and proinflammatory cytokines of neutrophil origin during mechanical tissue injury and severe infection. These are the conditions under which a transient increase in Lp(a) is observed in healthy individuals.

In addition, the pathogenic potential of Lp(a) is also affected by genetic factors. Three chromosomal regions, 9p21, 1p13, and 6q26-27, have been linked to Lp(a) production associated with CVD, but the *LPA* gene 6q26-27 has been shown to carry the greatest CV risk.

This is explained by the different structure of Lp(a) particles determined by the different expression of the *LPA* gene at the recognized sites. Lp(a) particles determined by the *LPA* gene from chromosome 6q26-27 have fewer KIV-2 fragment repeats in their structure, are smaller, and have a higher molecular density compared to Lp(a) fractions with a large number of KIV-2 fragment repeats [40].

## Lipoprotein(a) and cardiovascular diseases

Apart from new therapeutic principles, Lp(a) dyslipidemia has attracted the attention of the scientific community with broader use of genetic and epidemiological studies that documented a strong association of Lp(a) > 30 mg/dL (>75 nmol/L) with higher ASCVD risk. Although an absolute risk threshold is not yet generally accepted, an estimated 20%–25% of the world’s population has an Lp(a) level of 50 mg/dL or more, a level recognized by the European Atherosclerosis Society to confer higher CV risk [20, 41, 42].

Lp(a) levels above the 75th percentile multiply the risk for myocardial infarction and aortic valve stenosis, whereas higher levels (above 90th percentile) are associated with a higher risk for heart failure. The risk of CV mortality and ischemic stroke especially increased at very high levels (above 95th percentile) [43–45].

Modern clinical practice recognizes coronary artery calcium (CAC) scoring as a indicator for early atherosclerosis when early prevention is still possible. The advantages of this indicator are objectivity, low-cost diagnostics, interpretation, and testing simplicity. Positive CAC score (> 0) is a prognostic criterion for accelerated atherosclerosis, whereas CAC score of 0 has a negative prognostic value (99%) for the 10-year risk of developing CVD [46, 47].

Higher levels of Lp(a) are a causal risk factor for the expression of inflammatory genes and arterial calcification genes [41].

Mehta et al. demonstrated that Lp(a) level and CAC score are independently associated with an increased risk of developing ASCVD. Lp(a) level > 50 mg/dL and CAC score were independently associated with ASCVD risk, and an Lp(a)-by-CAC interaction was not found. On the other hand, high Lp(a) levels and CAC score, although characterized as independent risk factors, might have synergistic rather than additive effects on ASCVD risk, with a hazard ratio of nearly 5.

The reported study results are of great clinical importance because they lead the way to better screening for people without CVD but with a high Lp(a) level or CAC score. For patients with Lp(a) level > 50 mg/dL, the next step is CAC assessment and vice versa. For those with CAC ≥100, the Lp(a) level is the standard. This approach would benefit the screening of patients with risk factors and higher CVD risk and sort them into the secondary prevention level group with Lp(a) target therapy [20, 48].

Aortic valve stenosis is the most common valvular heart disease in developed countries. A sensitive technique to detect this disease in its early stages, even before clinical manifestations, is non-contrast CT [49–52]. Modern approaches to lower Lp(a) concentrations by up to 90% highlight the importance of lowering Lp(a) levels in people in the early stages of this disease. Namely, Kaiser et al. [53] presented the results of a comprehensive study demonstrating that aortic valve stenosis occurs in 1% of participants younger than 45 years and that the prevalence of this condition increases with age, occurring in 59.4% of people older than 80 years.

The same study confirmed previous findings that aortic valve calcification (AVC) is the first stage of aortic valve disease and that Lp(a) plays a role in the etiology of this disease. In the population aged 45–54 years, the expected incidence of aortic valve disease is 4.3%. However, in this population, individuals at high risk for AVC (15.3%) were identified, namely, those with Lp(a) levels above the 80th percentile, i.e., 50 mg/dL. It is of utmost importance to note that the association between Lp(a) and AVC was independent of age, sex, and other risk factors. According to the authors of the aforementioned study, it is precisely these individuals with Lp(a) levels > 50 mg/dL who would benefit most from non-contrast CT imaging and, in the case of AVC, from intensive treatment with drugs that effectively lower Lp(a) [53].

In addition, high Lp(a) levels have been associated with both micro- and macro-calcifications of the blood vessel walls and the aortic valve [54, 55]. High Lp(a) levels also lead to rapid progression of aortic stenosis, which in turn may result in the need for potential surgical valve replacement or a fatal outcome [56]. The effect of Lp(a) levels on atherosclerotic plaque progression has been well documented using coronary computed tomography angiography. While the non-contrast CT method addresses only the CAC score, this more complex angiographic method puts on display the state of both the calcified and non-calcified part of the plaque, which is of the utmost importance, given that Lp(a) is responsible for the progression of the non-calcified part [57, 58].

Using this precise technique, Kaiser et al. [53] demonstrated that Lp(a) levels correlate positively with volume progression of both calcified coronary artery plaques and fibrous plaques, independent of other risk factors. In this study, rapid plaque progression was documented within 12 months in individuals with cut-off values of Lp(a) ≥ 70mg/dL. The phenotypic plaque appears as microcalcified in individuals with elevated Lp(a) levels. It has a thin fibrous cap, is inflamed, and has an enlarged necrotic core, making it more prone to rupture and MACE.

Keynotes that were pointed out indicate that the use of statins and standard preventive measures for people at increased CV risk does not provide sufficient results in the prevention of plaque progression [57]. The results published by Kaiser et al. [53] match those documented and published only a few years earlier in the large, prospective, multinational ICONIC study (Incident Coronary Syndromes Identified by Computed Tomography) [59].

Patel et al. [60] estimated the risks of developing a CV event defined as myocardial infarction with acute complications, bypass or percutaneous coronary angioplasty/stent implantation, or ischemic stroke or cerebrovascular syndrome using a biobank of 500,000 subjects, people aged 40–69 years, with a median follow-up of 11.2 years, with corresponding use of statin therapy in subjects in primary and secondary prevention.

The study results indicated that higher Lp(a) concentration was detected in 12.2% of subjects without the abovementioned incidents. In comparison, an elevated Lp(a) concentration was detected in 20.3% of the patients with a CV incident. The group without a previous incident and with elevated Lp(a) levels had a 10-year risk of developing ASCVD of 4.8%, in contrast to the subpopulation whose Lp(a) concentration was below the cutoff value.

Compared with previous study results and in the group of subjects with a preexisting CV incident, the group with elevated Lp(a) concentration had a 10-year risk of developing a secondary event of 14.7%, in contrast to the subpopulation of those with lower Lp(a) levels, whose secondary risk was 12.9% [61].

During the lifespan of an average adult, there is no significant variation in Lp(a) levels. Variations of a purely physiological nature amount to about 10%. For this reason, it is advisable to screen high-risk patients only once, without further repeated Lp(a) level controls, unless medication therapy is added. In addition, no sex difference in Lp(a) levels was documented, but ethnic and population differences were noted. Namely, the lowest Lp(a) levels were found in white race subjects, while the highest levels were recorded in black subjects. However, the 80th percentile is set as the cutoff value, more specifically, a value above 50 mg/dL, regardless of race [27, 61–63].

According to the literature, screening for elevated Lp(a) levels is recommended in the following cases:

- premature CVD (CVD occurring before the age of 55 years in men and 60 years in women);
- family history of premature CVD;
- family history of elevated Lp(a) levels;
- recurrence of CV events despite LDL cholesterol levels in the reference range achieved with an antihyperlipidemic agent;
- ≥5% risk of an adverse CV event according to the Systematic Coronary Risk Evaluation (SCORE) assessment [15, 64].

In the pediatric population, screening is recommended in children with a history of ischemic stroke or in children whose parents have a history of ASCVD in the absence of other risk factors. In addition, repeated measurements of Lp(a) levels in children are recommended because Lp(a) levels increase during the growth and developmental period [44, 65, 66].

## Therapeutic options

Table 1 shows the currently widely used antihyperlipidemic therapeutic modalities and their effect on changes in Lp(a) levels.

**Table 1:** Antihyperlipidemic therapeutic modalities and their effect on lipoprotein(a)

	**Mechanism of action**	**Effects on Lp(a)**	**Testing phase**
ASOs (Pelacarsen)	Formation of mRNA-ASO complex and inhibition of mRNA	Reduction of apo(a) synthesis up to 95%	Lp(a) HORIZON-trial ongoing
siRNA (Olpasiran)	Inhibition of *LPA* mRNA gene synthesis	Reduction of apo(a) synthesis up to 98%	OCEAN(a)-trial ongoing

Lp(a): Lipoprotein(a); apo(a): Apoprotein(a); ASO: Antisense oligonucleotides; siRNA: Small interfering RNA.

In the absence of approved specific Lp(a)-lowering medications, the panel (ESC/EAS guidelines 2019) recommends early, intensive management of other CV risk factors (treatment of LDL cholesterol, arterial blood pressure, and glucoregulation) for individuals with elevated Lp(a) levels, which is consistent with most guidelines [66, 67].

Therapeutic measures used so far, including statin therapy and dietary measures, did not produce the desired outcome in patients with elevated Lp(a). On the contrary, a meta-analysis of six randomized-controlled studies of statins which included 5256 patients demonstrated an increase in Lp(a) levels of up to 11% [68, 69].

**Table 2:** Similarities and differences between apoprotein(a) inhibitors

**Therapeutic option**	**Primary indication**	**Effect on Lp(a)**
LDL-apheresis	HeFH, HFH, elevated LDL cholesterol level in secondary prevention of cardiovascular outcome	Reduction up to 60%
Statins	HeFH, HFH, elevated LDL cholesterol level in primary and secondary prevention of cardiovascular outcome	Increase by 11%
PCSK9 inhibitors	HeFH, HFH, elevated LDL cholesterol level in primary and secondary prevention of cardiovascular outcome	Reduction up to 20%–30%
Niacin and CETP inhibitors	HeFH, HFH, elevated LDL cholesterol levels, have no effect on cardiovascular outcomes	Reduction up to 20%–30%

Lp(a): Lipoprotein(a); LDL: Low-density lipoprotein; PCSK9: Proprotein convertase subtilisin/kexin type 9; HeFH: Heterozygous familial hypercholesterolemia; HFH: Homozygous familial hypercholesterolemia; CETP: Cholesterylester transfer protein.

In the same study, it has also been shown that the elevation of Lp(a) levels is both dose- and time-dependent, leading the authors to conclude that the longer statins are taken, the higher Lp(a) levels become. One possible explanation for this phenomenon is that statin therapy increases the expression of *LPA* mRNA, leading to increased apo(a) production. Nevertheless, according to the current guidelines for hypercholesterolemia management, statins are considered first-line therapy; however, their possible role in the residual CVD risk raises concern [68, 70].

An optimized diet with reduced fat intake and physical activity has not yet produced the desired results. Data from the literature suggest that a low-fat diet could increase Lp(a) levels. It has been shown that only a diet low in carbohydrates and rich in saturated fatty acids can reduce Lp(a) levels by up to 15% [25, 71, 72].

LDL apheresis has proven to be an effective therapeutic solution, since literature data and clinical experience suggest that it can reduce Lp(a) plasma levels by up to 60% [73]. Moreover, LDL apheresis has been shown to reduce the risk of major adverse CV events, defined as CV death, coronary bypass surgery, nonfatal myocardial infarction, percutaneous coronary intervention, or stenting, by up to 86%, as well as to reduce the annual risk of myocardial infarction by up to 97% [73, 74]. Additionally, because it is mostly apo(a) that contributes to the pathogenic potential of Lp(a), new therapeutic approaches focused on reducing the production of this apoprotein in liver hepatocytes are being explored.

A monoclonal antibody that inhibits PCSK9 has also been shown to be effective in lowering Lp(a) levels [75, 76]. The effect of PCSK9 inhibitors on Lp(a) levels is actually quite expected, considering that PCSK9 levels correlate with Lp(a) levels in the general population [77].

In a FOURIER trial, whose main objective was to determine the efficacy of the monoclonal antibody evolocumab in lowering LDL cholesterol and the risk of CV events in patients previously treated with statins, a subpopulation of patients with Lp(a) was also included. In this group, a 26.9% reduction in Lp(a) levels was achieved. The primary outcome, the incidence of CV death, myocardial infarction, stroke, hospitalization for coronary revascularization, or unstable angina, occurred in 12.6% of the evolocumab group vs 14.6% of the placebo group. This finding was consistent across all subgroups studied [75]. Recent studies with evolocumab demonstrated that combined triple therapy with statin/ezetimibe/PCSK9 inhibitor can reduce Lp(a) by 73.59% [78].

## Apo(a) inhibitors

Due to the fact that apo(a) is the main carrier of the pathogenic potential of Lp(a) and no therapeutic option, as shown in Table 1, has yet produced a satisfactory result in reducing Lp(a), interference with the mRNA of the *LPA* gene has been developed as a therapeutic option, the sole purpose of which is to inhibit the synthesis of apo(a) [36]. However, no hypolipidemic agent has yet been approved for the targeted treatment of patients with elevated Lp(a) levels.

Two subtypes of mRNA-interfering molecules have been developed. A representative of one subtype, antisense oligonucleotides, is Pelacarsen, and the other subtype, corresponding to small interfering RNA (siRNA), is olpasiran (Table 2) [36].

Pelacarsen belongs to the second generation of IONIS-APO(a)(Rx), i.e., the antisense oligonucleotides variant. IONIS-APO(a)(Rx) are DNA fragments about 16–20 nucleic acids long that are complementary to the mRNA of the *LPA* gene, which is responsible for the synthesis of apoprotein(s). After subcutaneous administration, IONIS-APO(a)(Rx) binds to a plasma protein, usually albumin, and then enters the extracellular space of the liver. From there, it enters the hepatocyte by various mechanisms. In hepatocytes, an mRNA-IONIS-APO(a)(Rx) complex is formed that inhibits the synthesis of apoprotein(s). Despite its structural similarity to plasminogen mRNA, IONIS-APO(a)(Rx) itself does not bind to the mRNA responsible for plasminogen synthesis. In this way, the synthesis of Lp(a) is inhibited, but the homeostasis of coagulation and fibrinolysis remains intact, which is certainly another important therapeutic target, since one of the pathogenetic mechanisms of elevated Lp(a) levels is to all intents, inhibited fibrinolysis [79].

Currently, pelacarsen (IONIS-APO(a)(Rx) is the focus of numerous clinical trials with high expectations regarding its Lp(a)-lowering potential. In phase I/II clinical trial, this medication showed a significant, dose-dependent reduction in Lp(a) levels. The highest dose administered, 20 mg/once weekly, lowered basal levels of Lp(a) by up to 80% [80–82]. A dose-dependent reduction in phospholipids of up to 90% and a reduction in LDL cholesterol of 20% were also registered in subjects who had already used statin therapy [83].

The phase III clinical trial evaluating the efficacy of pelacarsen and assessing the 5-year cardiovascular outcomes, called “Assessing the Impact of Lipoprotein(a) Lowering with Pelacarsen (TQJ230) on Major Cardiovascular Events in Patients with CVD (Lp(a) HORIZON)” is still ongoing. Eight thousand three hundred and twenty-four patients (8324) have been enrolled in the trial, and the first results are expected to be published in May 2025 [84].

Olpasiran is a first-in-class, synthetic, double-stranded, N-acetylgalactosamine-conjugated small interfering RNA (siRNA) designed to directly inhibit translation of *LPA* mRNA in hepatocytes and effectively reduce plasma Lp(a) concentration [84–86]. Evaluation of olpasiran efficacy has shown that it could reduce Lp(a) levels by 71%–97% [85].

Another study evaluating the efficacy of olpasiran is also ongoing. OCEAN(a)-DOSE trial (The Olpasiran trials of Cardiovascular Events And lipoproteiN(a) reduction-DOSE finding study) is a multicenter, randomized, double-blind, placebo-controlled dose-finding study, which enrolled 281 participants with established ASCVD and Lp(a) > 150 nmol/L. The primary goal is to determine and evaluate the effects of olpasiran administered every 12 weeks compared to placebo on the percent change from baseline in Lp(a) at 36 weeks. These data will be used to determine the optimal dosing and design for a CV outcomes trial [87].

## Limitations

The prognostic value of Lp(a) levels in different racial and ethnic groups remains unclear. Although previous genetic and epidemiological research that has been conducted so far concluded that African Americans have higher Lp(a) levels, Patel et al. [60] found no difference in CV outcome based on Lp(a) levels between patients of different ethnic groups [88].

## Conclusion

In conclusion, the authors of this article would like to highlight that there are not enough randomized controlled trials assessing the CV outcomes that would support the evidence that current therapy options that are effective in lowering Lp(a) levels are also effective in preventing CV events. However, according to some studies, there is strong evidence that better CV outcomes are among the benefits of such therapy. These facts are to be expected because PCSK9 inhibitors and pelacarsen have only recently been used. Therefore, which is why the results of the Lp(a)HORIZON trial, which is still ongoing, are eagerly awaited.
